# Novel Insights on the Regulation of B Cell Functionality by Members of the Tumor Necrosis Factor Superfamily in Jawed Fish

**DOI:** 10.3389/fimmu.2018.01285

**Published:** 2018-06-07

**Authors:** Carolina Tafalla, Aitor G. Granja

**Affiliations:** Animal Health Research Center (CISA-INIA), Madrid, Spain

**Keywords:** TNFSF, TNFRSF, fish, B cells, evolution, immunity

## Abstract

Most ligands and receptors from the tumor necrosis factor (TNF) superfamily play very important roles in the immune system. In particular, many of these molecules are essential in the regulation of B cell biology and B cell-mediated immune responses. Hence, in mammals, it is known that many TNF family members play a key role on B cell development, maturation, homeostasis, activation, and differentiation, also influencing the ability of B cells to present antigens or act as regulators of immune responses. Evolutionarily, jawed fish (including cartilaginous and bony fish) constitute the first animal group in which an adaptive immune response based on B cells and immunoglobulins is present. However, until recently, not much was known about the expression of TNF ligands and receptors in these species. The sequences of many members of the TNF superfamily have been recently identified in different species of jawed fish, thus allowing posterior analysis on the role that these ligands and receptors have on B cell functionality. In this review, we summarize the current knowledge on the impact that the TNF family members have in different aspects of B cell functionality in fish, also providing an in depth comparison with functional aspects of TNF members in mammals, that will permit a further understanding of how B cell functionality is regulated in these distant animal groups.

## Introduction

In mammals, the tumor necrosis factor (TNF) ligand superfamily (TNFSF) signal through members of the tumour necrosis factor receptor superfamily (TNFRSF) to activate signaling pathways which play biological roles in development, organogenesis, cell death, and survival. Since the discovery of the first TNFSF members, TNF (1) and lymphotoxin α (Ltα) (2), more than 40 years ago, 17 additional TNFSF members and 29 cognate receptors have been identified in humans (3). With the completion of the large-scale sequencing of the human and mouse genomes, it is assumed that almost all TNFSF and TNFRSF members have now been identified in mammals (3, 4). Thus, ligands within the TNFSF which include 4-1BBL, a proliferation-inducing ligand (APRIL), B cell-activating factor of the TNF family (BAFF), CD27L, CD30L, CD40L, EDA1, EDA2, Fas ligand (FasL), GITRL, LIGHT, Ltα, Ltβ, OX40L, RANK ligand, TL1A, TNF, TNF-like weak inducer of apoptosis and proliferation, and TNF-related apoptosis-inducing ligand (TRAIL) are key effector proteins in the orchestration of innate and adaptive immune responses [reviewed in Ref. (3) and summarized in Table 1].

**Table 1:** Relation of TNF superfamily ligands (TNFSF) present in human indicating their standard name within the TNF superfamily and their alternative (most common) name.

Standard name (encoding gene)	Alternative name	UniProt ID
TNFSF1	Ltα	P01374
TNFSF2	TNF-α	P01375
TNFSF3	LTβ	Q06643
TNFSF4	OX40L	P23510
TNFSF5	CD40L	P29965
TNFSF6	FasL	P48023
TNFSF7	CD70	P32970
TNFSF8	CD153	P32971
TNFSF9	4-1BB-L	P41273
TNFSF10	TRAIL	P50591
TNFSF11	RANKL	O14788
TNFSF12	TWEAK	O43508
TNFSF12-13	TWE-PRIL	O43508-2
TNFSF13	APRIL	O75888
TNFSF13b	BAFF	Q9Y275
TNFSF14	LIGHT	O43557
TNFSF15	TL1A	O95150
TNFSF18	GITRL	Q9UNG2
N/A	EDA	Q92838

*Uniprot ID links for each ligand are also provided*.

TNFSF ligands are type II membrane-bound proteins with an intracellular N terminal and an extracellular C terminal domain. Among the 19 known ligands described in human and mouse, 11 encode for a proteolytic cleavage site that generates biologically active soluble forms (5). Within the C terminus, they contain the TNF homology domain (THD), which presents a weak degree of conservation (20–30%) between ligand members, and is typically formed by 10 β-strands (6). The THD folds into what has been called an antiparallel β-sandwich (3) exhibiting a compact jellyroll topology (4). The THD structure has the ability to assemble each TNF ligand into conical trimers which allow the ligands to bind respective receptors to initiate signaling (6).

To date, 29 TNFRs have been identified in mammals, namely, 4-1BB, BAFFR, B cell maturation antigen (BCMA), CD27, CD30, CD40, decoy receptor 3 (DcR3), death receptor 3, DR6, EDAR, Fas, Fn14, glucocorticoid-induced TNFR, herpesvirus entry mediator (HVEM), LTbR, OPG, OX40, RANK, receptor expressed in lymphoid tissues (RELT), transmembrane activator and calcium-modulating cyclophilin ligand interactor (TACI), TNFR1, TNFR2, TRAILR1, TRAILR2, TRAILR3, TRAILR4, TROY, and XEDAR [reviewed in Ref. (7)]. The main feature of these TNFRs is a cysteine-rich domain (CRD) formed of three disulfide bonds surrounding a core motif of CXXCXXC creating an elongated molecule. There is an important variation in the number of CRDs among family members, from BAFFR or BCMA containing only one CRD to CD30 containing six CRDs. These receptors are type I membrane proteins, with the exceptions of BAFFR, BCMA, TACI, and XEDAR, which are type III membrane proteins, and OPG and DcR3, which are secreted (4, 8). The determination of the X-ray crystal structures of some ligands bound to the extracellular domain of their receptors has been primordial to characterize the binding mechanisms between them (9, 10). These analyses have revealed that those receptors with several CRDs adopt an elongated structure and bind at the interface between two ligand monomers, whereas single CRD receptors are more compact and contact a single ligand monomer in a trimeric ligand (5, 11, 12). Generally, one trimeric ligand engages three monomeric receptors, a key event for the activation of intracellular signaling pathways. By conducting a systematic flow cytometry-based assay, Bossen et al. elegantly demonstrated the mechanisms that regulate TNFSF–TNFRSF interactions in mouse and human (8). They found that TNFSF ligands bound from one to five different receptors, while most receptors bound from one to three ligands. Strikingly, they observed that, although containing the classical CRD structure, DR6, RELT, TROY, and nerve growth factor receptor (NGFR) did not bind to any of the TNFSF, thus suggesting that they either bind to other ligands or function in a ligand-independent manner. In this sense, it was later shown that although NGFR has the classic CRD structure, it binds a structurally different kind of ligands, the neurotrophins (13). This information regarding the different ligands that signal through each receptor in mammals is summarized in Table 2.

**Table 2:** Functional relation of the tumour necrosis factor receptor superfamily (TNFRSF) and their cognate ligands (TNFSF).

Name	Gene	Ligands	Immune functions	Reference
LTβR	Tnfrsf3	LTβ	Development and organization of lymphoid tissues	(14)
TNFR1	Tnfrsf1A	LTα, TNF-α	Development of lymphoid tissues B and T cell activation	(15)
TNFR2	Tnfrsf1B	LTα, TNF-α	T cell activation	
Fn14	Tnfrsf12	TWEAK	Inflammation Angiogenesis	(16)
HVEM	Tnfrsf14	LTα, LIGHT	T cell homeostasis	(17)
DcR3	Tnfrsf6B	FasL, LIGHT, TL1A	Inhibition of FasL and LIGHT mediated apoptosis of T cells	(18)
Fas	Tnfrsf6	FasL	T cell homeostasis (death) T cell co-stimulation	(19)
GITR	Tnfrsf18	GITRL	T cell survival and activation	(20)
CD40	Tnfrsf5	CD40L (CD154)	T cell-mediated activation of B cells and dendritic cells (DCs)	(21)
OX40	Tnfrsf4	OX40L	B and T cell activation	(22)
TRAILR1	Tnfrsf10A	TRAIL	T cell and natural killer cell-mediated tumor surveillance Apoptosis of tumoral cells Regulation of T cell functions	(23)
TRAILR2	Tnfrsf10B			
TRAILR3	Tnfrsf10C			
TRAILR4	Tnfrsf10D			
RANK	Tnfrsf11A	RANKL	B cell and DC maturation	(24)
OPG	Tnfrsf11B	RANKL TRAIL	Lymph node formation Bone homeostasis	(25)
BAFFR	Tnfrsf13C	BAFF	B cell maturation and survival	(26)
BCMA	Tnfrsf17	BAFF, APRIL	Plasma cell survival	(27)
TACI	Tnfrsf13B	BAFF, APRIL	T-independent B cell responses	(28)
CD27	Tnfrsf7	CD70	B cell and T cell activation	(29)
4-1BB	Tnfrsf9	4-1BB-L	T cell survival and activation	(30)
CD30	Tnfrsf8	CD153	B cell and T cell activation	(31)
TROY	Tnfrsf19	Unknown	Development of ectoderm-derived tissues	(32)
EDAR	Edar	EDA-A1		
XEDAR	Xedar	EDA-A2		
DR3	Tnfrsf25	TL1A	T cell co-stimulation	(33)
DR6	Tnfrsf21	APP	Induction of neuronal death	(34)
RELT	Tnfrsf19L	Unknown	Co-stimulation of T cells	(35)

*TNFRSF containing a death domain are underlined. Main immune functions known for each TNFRSF are also indicated, together with the references describing their functions in mammals*.

## Evolution of TNFSF and TNFRSF Members

The appearance and further specialization of the adaptive immune response is a hallmark of the successful evolution of vertebrates. The adaptive immune system is based on the presence of recombination-activating gene (RAG)-recombined B cell receptors (BCR) and T cell receptors (TCR) on the surface of B cells and T cells, respectively, and the major histocompatibility complex (MHC). Molecular studies have shown that adaptive immunity arose early on vertebrate evolution, between the divergences of cyclostomes (lampreys) and cartilaginous fish, around 450 million years ago, by diversification and recombination of gene clusters on a span of time of 20 million years (36). This event is known as the “big bang” theory of the appearance of the adaptive immune response, since it occurred in the very short period of time in which jawed fish appeared and is thought to be linked to genome duplication events (37). Thus, jawed fish were the most ancient animal group where all these elements were found, while jawless fish (Agnathans) seemed to have none of them (38). However, the posterior discovery of a lymphoid cell-based adaptive immune system in Agnathans, in which immune receptors recombined in a similar way to that of the BCR (39), pushed the origin of the adaptive immune response earlier in evolution. Despite this, the “big bang” theory for the origin and development of acquire immunity still prevails.

Interestingly, Collette et al. postulated that the divergence of the TNFSF and TNFRSF members parallels the emergence of the adaptive immune response (7). Since 11 out of the 19 human TNFSF members are clustered within the MHC and paralogous regions on chromosomes 1, 6, 9, and 19, the authors suggest that this disposition might be a consequence of the ancestral arrangement of a proto-TNFSF cluster, before en bloc duplication of the proto-MHC region in a vertebrate ancestor 500–800 million years ago (40). Remarkably, the different number of TNFSF and TNFRSF members found in the different species within vertebrates correlates with the number of rounds of genome duplication during evolution (4), thus supporting the idea that genome duplication created paralogous clusters. This hypothesis is further supported by phylogenetic analysis that indicates an ancient evolutionary origin of TNFSF ligand and receptor genes that precedes the appearance of vertebrates (7, 41).

As many invertebrate and vertebrate genomes are now available, the discovery of TNFSF and TNFRSF orthologs and paralogs has greatly increased. Consequently, recent phylogenetic studies on invertebrate TNFSF ligands and receptors have been key to better understand the appearance of TNF molecules in metazoans and to further support the hypothesis of their divergent evolution [reviewed in Ref. (4)]. The most primitive TNF superfamily member that has been functionally characterized is Eiger, a TNFSF homolog found on the fruit fly (*Drosophila melanogaster*) (42). Eiger binds to a cognate TNFRSF member, called Wengen, which contains an intracellular death domain, thus inducing cell death through the activation of signaling pathways similar to those activated by mammalian TNFRSFs (43, 44). A TNFSF member named MjTNF has also been characterized in another invertebrate, the marine arthropod kuruma shrimp (*Marsupenaeus japonicas*) (45). This protein contains a predicted transmembrane region and a THD, and shares 30.7% sequence identity with *Drosophila* Eiger. Two molluscan TNFSF members containing transmembrane regions and THDs were identified in the disk abalone, *Haliotis discus discus*. One was designated AbTNF-α (46) and the other AbFas ligand (47). Within the genome of the equinoderm purple sea urchin (*Strongylocentrotus purpuratus*), four different TNFSF genes have been found, identified as potential gene orthologs of TNFSF14 (LIGHT), TNFSF15 (TL1A), and two separate genes resembling EDA (48, 49). In parallel, several TNF superfamily receptors were also identified on these invertebrate organisms [summarized in Ref. (4)]. As most of the invertebrate TNFSF members are constitutively expressed and most TNFRSFs are phylogenetically related to EDAR, a major role in development and organogenesis with restricted immunoregulatory properties is foreseen in these organisms.

By contrast, in teleost fish, the first animal group comprising all the elements of the adaptive arm of the immune system, a diversification of the TNFSF and TNFRSF has occurred. To date, 13 different TNFSF and 13 TNFRSF homologs have been identified, together with new members of the TNFSF that are novel to this animal group (4, 41, 50–54) supporting the hypothesis of a diversification of the TNF superfamily with the appearance of the adaptive immune response. Interestingly, recent studies in sarcopterygian fish (African lungfish) revealed a different TNFSF and TNFRSF gene pattern to that seen in teleost (55). African lungfish is an extant representative of the closest ancestral lineage to all tetrapods that presents organized lymphoid structures which cannot be found in other fish species (56). Several TNFSF were reported in this study, but additional analyses are needed to further characterize their identity and functionality. In parallel, many TNFRSF members were identified, and although most of them were present in both teleost and lungfish, only the latter presented homologue sequences for the TNF receptors HVEM (TNFRSF14), 4-1BB (TNFRSF9), and OPG (TNFRSF11B) (55). These TNF superfamily members have been shown to regulate T cell homeostasis and activation, as well as development of lymphoid structures, such as lymph nodes (LNs) (15, 17, 25, 30, 31). These results suggest that an expansion of TNF molecules could have occurred in the African lungfish, conferring a phenotypical advantage that was positively selected, thus leading to the appearance of organized lymphoid structures, which might play a key role on T cell activation. Therefore, throughout the evolution of vertebrates, the expansion of TNFSF and TNFRSF has led to the appearance of new members involved in the regulation of novel immune functions (4, 57), which were coopted under selective pressure, being this crucial for the evolution of the adaptive immune system (7, 41).

## Role of TNFSF Ligands on B Cell Regulation

**Figure 1:**
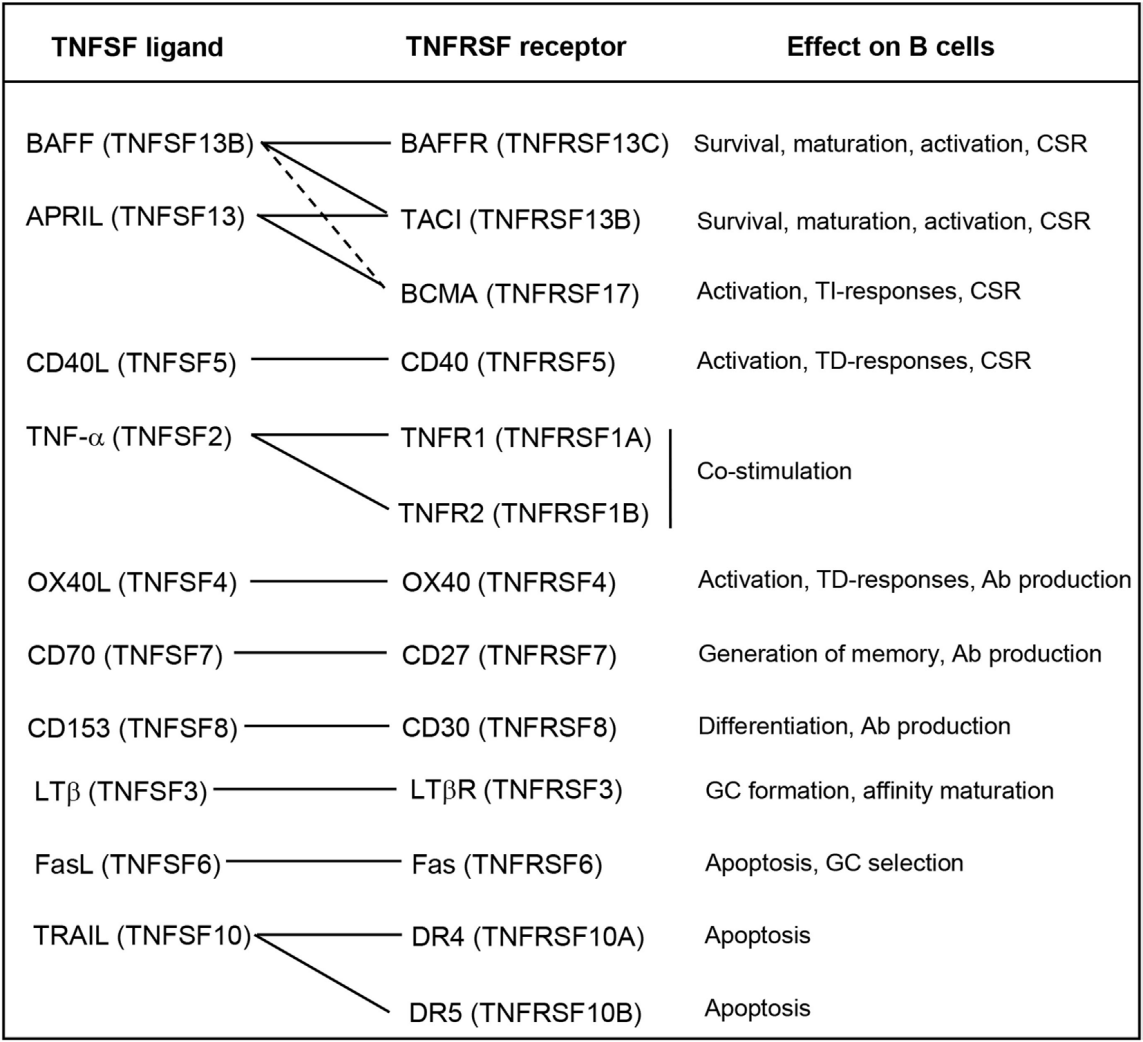
Roles of the TNFSF on B cell functions. Known interactions between ligands and receptors are indicated with solid lines, or dashed lines if the interactions are of weak affinity. Abbreviations: Ab, antibody; CSR, class-switch recombination; GC, germinal center; TD, thymus dependent; TI, thymus independent.

In mammals, many TNFSF ligands have been shown to play essential roles on the regulation of the functionality of B cells. These TNFSF ligands may influence all aspects of B cell biology from development, maturation, survival, proliferation, activation, and differentiation [reviewed in Ref. (58)] (Figure 1), thus playing a fundamental role on B cell-mediated immune responses.

Among TNFSF members, BAFF (TNFSF13B) and APRIL (TNFSF13) are probably the cytokines that seem to play a prevailing role on the regulation of B cell activity [reviewed in Ref. (59)]. These two cytokines exist as membrane-bound and soluble forms, being both forms biologically active (60). Both BAFF and APRIL bind to and signal through BCMA (TNFRSF17) and TACI (TNFRSF13B), whereas BAFF also binds to BAFFR (TNFRSF13C). These receptors are mainly expressed in B cells, and their specific activation leads to different outcomes of the B cell response (61). BAFF-mediated survival signals through BAFFR are necessary for immature B cells to become mature circulating B cells and for peripheral B cell survival (62). These signals regulate the size of the B cell compartment, especially that of conventional B2 cells, since the absence of BAFF does not affect the maturation or survival of innate-like B cells, such as marginal zone (MZ) or B1 cells (63). In fact, there is some evidence to suggest that the maintenance of the B1 B cell compartment is controlled by APRIL signaling through TACI (64, 65), which is highly expressed on the surface MZ B cells and B1 cells (66). In this context, BAFF and APRIL signaling through TACI have been shown to induce class-switch recombination (CSR) in response to thymus-independent (TI) antigens (Ags) (28).

Other TNFSF members can induce CSR on B cells in response to thymus-dependent (TD) Ags; such as, for example, CD40L (TNFSF5) and OX40L (TNFSF4) (21, 22). CD40L is expressed mainly by activated T cells during TD responses, thus mediating the co-stimulation of BCR-activated B cells, which express the receptor CD40 (TNFRSF5), usually within the germinal center (GC). As a consequence, co-stimulated B cells in the GC enhance their proliferation and undergo somatic hypermutation (SHM) to increase their affinity and CSR to switch from producing IgM to producing immunoglobulin isotypes with higher Ag affinity such as IgA, IgE, or IgG [reviewed in Ref. (21)]. In this context, part of these CD40L-induced proliferating B cells also differentiates to antibody (Ab)-secreting plasma cells (PCs), since CD40L and IL-21 synergistically induce the expression of B lymphocyte-induced maturation protein-1 (67), a transcription factor which is the master regulator of terminal differentiation to PCs (68). Moreover, BAFF and APRIL signaling through BAFFR and TACI can contribute to enhance PC differentiation triggered by CD40L (69). On terminally differentiated PCs, signaling through BCMA is highly expressed on PCs and is needed to promote their survival (70). Both BAFF and APRIL can signal through BCMA, although it shows much higher affinity for APRIL than BAFF (71). Concerning OX40L, this cytokine is expressed mainly on activated B cells while its receptor OX40 is expressed on activated CD4^+^ T cells. Their interaction triggers a bidirectional co-stimulation of both B and T cells during TD responses. Furthermore, cross-linking of OX40L on B cells by OX40 has been shown to greatly enhance B cell proliferation and Ig production (22).

One of the most studied TNFSF members is TNF-α, a well-known pro-inflammatory cytokine able to promote cell death (72). However, TNF-α has also been shown to play an important role on B cell functionality (73). TNF-α binds to two different receptors, TNFR1, which is ubiquitously expressed on almost all cell types, and TNFR2, whose expression is limited to the central nervous system and the immune system, especially found on T cells (15). TNF-α expression is rapidly and strongly upregulated *in vitro* or *in vivo* in the presence of many types of Ags or inflammatory mediators (15). In addition, TNF-α is produced by T cells after TCR engagement (74) and by B cells after TI BCR cross-linking and also after CD40 ligation by T cell-derived CD40L (75). In this context, TNF-α provides co-stimulatory signals which increase the proliferation and Ab production of B cells after Ag encounter, being very important for the polyclonal expansion needed within primary responses (15).

After BCR engagement, expression of CD70 (TNFSF7) is also induced on B cells. Ligation of CD70 with its ligand CD27 delivers signals to enhance proliferation, inhibit B cell differentiation to PCs, trigger SHM, and promote the generation of memory B cells (76). However, it has also been shown that ligation of CD70 in the presence of co-stimulatory T cell signals such as CD40L can promote B cell differentiation into Ab-producing PCs (77).

Recent studies have shown that BCR cross-linking increases the sensitivity of B cells to TRAIL (TNFSF10)-mediated cell death. It has been demonstrated that this effect can be reverted by ligation of CD40 on B cells, while B1 cells, which are involved in TI responses showed very high sensitivity to TRAIL-induced death. These data suggested that TRAIL is involved in B cell differentiation and survival at the GC reaction, and in Ab affinity maturation (78). Another member playing a similar role is Fas ligand (FasL) (TNFSF6), which induces apoptosis after ligation of its receptor (Fas) on the surface of the target cell (79). BCR activation induces the expression of Fas on the surface of B cells, making them more susceptible of FasL-mediated apoptosis. During the GC reaction, CD40 ligation protects B cells from Fas-induced apoptosis, thus contributing to the selection of B cells bearing a high-affinity BCR (80). LTβ has also been demonstrated to play an important role in the formation of GCs and also on Ab affinity maturation (81). Finally, CD153 (TNFSF8) also plays a role on B cells since the binding to its receptor (CD30) on T cells modulates B cell differentiation and CSR mediated by reverse signaling induced by CD30^+^ activated T cells (82).

## The Adaptive Immune System in Fish

The adaptive immune system, characterized by an Ag-specific combinatorial immune response (36), first appeared in jawed fish. Thus, evolutionarily, cartilaginous fish (sharks, skates, and rays) are the first animal group in which the adaptive immune system, based on immunoglobulin superfamily members, namely, BCR, TCR and MHC, and RAG 1 and 2 genes are present (38).

Due to the anatomical differences between fish and mammals (i.e., humans), significant differences are found in the distribution and functionality of primary and secondary lymphoid organs, such as the absence of LN or bone marrow (BM) in fish (56, 83). The fish spleen functions as the major secondary lymphoid organ, as it happens in mammals, and since fish lack LN, the spleen has been shown as the most important tissue for Ag trapping (84).

Regarding hematopoiesis, fish do not have a conventional BM as it is described in the mammalian immune system. In cartilaginous fish, the Leydig organ and the epigonal organ are believed to be the equivalents of mammalian BM (85). Both are reticular structures that contain large numbers of immature leukocytes, including neutrophils, eosinophils, and other granulocytes, as well as lymphocyte aggregates with scattered PCs. Either one or both of these tissues have been demonstrated to be present in all cartilaginous species examined (83). The expression of RAG-1 and B-cell-specific transcription factors strongly supports a lymphopoietic role for these tissues (86). In the case of bony fish (teleost), the anterior part of the kidney (head kidney/anterior kidney) has no renal functions and has been shown to assume hematopoietic functions (87). B cell development at the anterior kidney has been proven by the expression of RAG-1/2 (88, 89), TdT (90), and the transcription factor Ikaros (91), and the posterior cellular analysis defining the B cell subsets residing within the kidney (92). As in mammalian BM (93), anterior kidney also stores Ig-secreting long-lived PCs (94).

The thymus is similar to that found in mammals, composed by a cortex and a medulla, and is responsible for the production of T cells (95). TCR cell surface expression has been shown in teleost (96) but remains to be described in cartilaginous fish. However, the expression of all the TCR genes identified in mammals (α, β, δ, and γ) has been reported in cartilaginous fish (97).

## Fish B Cells

B cells are one of the most important elements of the adaptive immune response, since they are able to produce specific high-affinity immunoglobulins against pathogens (Abs), and also generate memory B cells which will protect the organism against future infections (98). In fish, there are important differences concerning the isotypes of immunoglobulins produced by B cells in comparison to mammals.

Cartilaginous fish B cells produce three types of immunoglobulins IgM, IgW, and IgNAR (immunoglobulin new antigen receptor) (38). In these species, IgM is orthologous to mammalian IgM, while IgW has been postulated as the orthologous of mammalian IgD (99), although its function is still unknown. IgNAR is a shark-specific heavy chain (H) homodimer which does not associate with light chains (L) (100). IgNAR is produced by a different B cell subset than that expressing IgM and although the specific function for IgNAR remains unclear, IgNAR responses have been shown to be TD and show high specificity for the Ag (101).

Teleost fish species produce three types of Igs, namely, IgM, IgD, and IgT/Z (102). The latter was first described in 2005 in both rainbow trout (*Oncorhynchus mykiss*) (designated as IgT for teleost) (103) and in zebrafish (*Danio rerio*) where it was designated as IgZ (104). Since then, it has been described in most teleost species (105, 106), while it seems absent in others such as channel catfish (*Ictalurus punctatus*) and medaka fish (*Oryzias latipes*) (107). While the most abundant B cell type in the main lymphoid organs in teleost is IgD^+^IgM^+^, as it has been described for mammalian naïve mature B cells (108), IgT^+^ B cells constitute a different lineage in teleost, which do not co-express any other surface Ig (109). These cells are more abundant in mucosal surfaces than in the main lymphoid organs and consequently IgT-expressing B cells have been cataloged as B cells specialized in mucosal responses (109–111). Despite this, IgT responses have also been reported outside the mucosal compartments, thus the function of IgT in teleost immune responses is still largely unknown. In addition, IgD^+^IgM^−^ (IgD single) cells have been identified in rainbow trout gills (112) and channel catfish blood (113) although their function is still unknown. Moreover, IgD^−^IgM^+^ (IgM single) cells exhibiting an antibody-secreting cell (ASC) phenotype have been shown to inhabit in the peritoneal cavity of vaccinated rainbow trout (114). These observations suggest that the B cell compartment in fish is composed by different cell subsets which most probably differ in functionality and/or cytokine-mediated regulation.

Interestingly, teleost B cells have been shown to possess strong phagocytic and microbicidal activities (115). It was later shown that is ability is preserved in mammalian innate B1 cells from the peritoneal cavity of mice (116) strongly suggesting that fish B cells are less evolved than mammalian B2 cells and still retain features of the innate immune system, which could be consistent with a posterior appearance of specific B1 and B2 B cell lineages throughout evolution. Further evidence supporting this hypothesis has been collected from studies showing the capacity of fish B cells to respond to pro-inflammatory stimuli (117), the expression of innate B cell markers CD9 and CD63 (118) and toll-like receptors (TLRs) (119) or the synthesis of antimicrobial peptides (117).

## TNFSF Ligands in Fish: At the Dawn of Acquired Immunity?

As mentioned earlier, Collette et al. proposed that the divergence of the TNFSF and TNFRSF families parallels the emergence of the adaptive immune system, after performing a detailed analysis of the phylogenetic relations between the TNFSF and TNFRSF members of invertebrate and vertebrate species (7). As the identification of TNFSF orthologs and paralogs in fish has been rapidly increasing in the recent years, as can be inferred from the work undertaken by Glenney and Wiens (4, 41), studying the function of these cytokines and their receptors in fish is becoming a fascinating research topic to better understand the evolution and regulation of the adaptive immune system. In this review, we illustrate the information available regarding the effect of TNFSF ligands in fish, focusing specifically on those with a presumed role on fish B cells.

## B Cell-Activating Factor of the TNF Family

The homologue sequences to mammalian BAFF have been reported in many teleost fish species including rainbow trout (41), zebrafish (120), mefugu (*Takifugu obscurus*) (121), Japanese sea perch (*Lateolabrax japonicus*) (122), grass carp (*Ctenopharyngodon idella*) (123), yellow grouper (*Epinephelus awoara*) (124), miiuy croaker (*Miichthys miiuy*) (125), tongue sole (*Cynoglossus semilaevis*) (126), Nile tilapia (*Oreochromis niloticus*) (127), rock bream (*Oplegnathus fasciatus*) (128), and also in cartilaginous fish such as white-spotted catshark (*Chiloscyllium plagiosum*) (129), spiny dogfish (*Squalus acanthias*) (130), and small-spotted catshark (*Scyliorhinus canicula*) (131) (summarized in Table 3). Interestingly, some studies have revealed that many cartilaginous and bony fish species have more than one BAFF gene (51, 54), representing two distinct groups. In mammals, BAFF is considered the master regulator of B cell development and function. Besides having been identified in many cartilaginous and bony fish species, it has been found in all vertebrate groups including birds, amphibians and reptiles (132), suggesting that BAFF has been essential throughout evolution for the development of peripheral mature B cells.

**Table 3:** B cell-regulating TNFSF ligand members identified to date in fish.

TNFSF ligand	Species (common name)	Gene name	Reference
BAFF (TNFSF13b)	*Oncorhynchus mykiss* (rainbow trout)	OmBAFF	(41)
	*Tetraodon nigroviridis* (spotted green pufferfish)	Tn_BAFF	(41)
	*Danio rerio* (zebarfish)	zBAFF	(120)
	*Takifugu obscurus* (mefugu)	fBAFF	(121)
	*Chiloscyllium plagiosum* (white-spotted catshark)	CpBAFF	(129)
	*Lateolabrax japonicus* (Japanese sea perch)	LjBAFF	(122)
	*Squalus acanthias* (spinny dogfish)	SaBAFF	(130)
	*Ctenopharyngodon idella* (grass carp)	gcBAFF	(123)
	*Epinephelus awoara* (yellow grouper)	EaBAFF	(124)
	*Miichthys miiuy* (miiuy croaker)	MmBAFF	(125)
	*Takifugu rubripes* (Japanese pufferfish)	FuguBAFF1 FuguBAFF2	(133)
	*Scyliorhinus canicula* (small-spotted catshark)	ScBAFF	(131)
	*Cynoglossus semilaevis* (tongue sole)	CsBAFF	(126)
	*Oreochromis niloticus* (Nile tilapia)	tBAFF	(127)
	*Oplegnathus fasciatus* (rock bream)	RbBAFF	(128)

APRIL (TNFSF13)	*O. mykiss* (rainbow trout)	Om_APRIL	(41)
	*Salmo salar* (Atlantic salmon)	Ss_APRIL	(41)
	*Ictalurus punctatus* (channel catfish)	Ip_APRIL	(41)
	*D. rerio* (zebrafish)	Cr_APRIL	(41)
	*C. idella* (grass carp)	gcAPRIL	(134)

BAFF- and APRIL-like molecule	*O. mykiss* (rainbow trout)	Om_BALM	(41)
	*T. nigroviridis* (spotted green pufferfish)	Tn_BALM	(41)
	*T. rubripes* (Japanese pufferfish)	Fr_BALM	(41)
	*Gasterosteus aculeatus* (three spine stickleback)	Ga_BALM	(41)

CD40L (TNFSF5)	*O. mykiss* (rainbow trout)	Om_CD40L	(41)
	*S. salar* (Atlantic salmon)	Ss_CD40L	(41)
	*D. rerio* (zebrafish)	Cr_CD40L	(41)
	*T. nigroviridis* (spotted green pufferfish)	Tn_CD40L	(41)
	*T. rubripes* (Japanese pufferfish)	Fr_CD40L	(41)
	*G. aculeatus* (three spine stickleback)	Ga_CD40L	(41)
	*S. canicula* (small-spotted catshark)	ScCD40L	(131)

OX40L (TNFSF4)	N/A		

TNF-α (TNFSF2)	*Paralichthys olivaceus* (Japanese flounder)	Japanese flounder TNF	(135)
	*O. mykiss* (rainbow trout)	OmTNF1 OmTNF2 TNFα3	(136–138)
	*Sparus aurata* (gilthead seabream)	Seabream TNF-α	(139)
	*I. punctatus* (channel catfish)	Catfish TNF-α	(140)
	*Cyprinus carpio* (common carp)	Carp TNF-1α Carp TNF-2α Carp TNF-3α	(141, 142)
	*Psetta maxima* (turbot)	Turbot_TNF-α	(143)
	*Dicentrarchus labrax* (sea bass)	sb TNF-α	(144)
	*Siniperca chuatsi* (mandarin fish)	Mandarin fish TNF-α	(145)
	*S. salar* (Atlantic salmon)	TNF-α1 TNF-α2	(146)
	*Carassius auratus* (goldfish)	TNF-α1 TNF-α2	(147)
	*Latris lineata* (striped trumpeter)	Striped trumpeter TNF-alpha	(148)
	*Thunnus orientalis* (bluefin tuna)	TNF1 TNF2	(149)
	*C. semilaevis* (tongue sole)	CsTNF1	(150)

CD70 (TNFSF7)	N/A		

CD153 (TNFSF8)	N/A		

LTβ (TNFSF3)	*D. rerio* (zebrafish)	TNF-N	(142)
	*T. rubripes* (Japanese pufferfish)	TNF-N	(142)
	*O. mykiss* (rainbow trout)	Trout LT-β1 Trout LT-β2	(50)
	*Oryzias latipes* (medaka)	Tnf-n	(151)

FasL (TNFSF6)	*D. rerio* (zebrafish)	zFasL	(152)
	*O. mykiss* (rainbow trout)	Om_FasL	(41)
	*T. rubripes* (Japanese pufferfish)	Fr_FasL	(41)
	*T. nigroviridis* (spotted green pufferfish)	Tn_FasL	(41)
	*P. olivaceus* (Japanese flounder)	FasL	(153)
	*O. niloticus* (Nile tilapia)	tFasL	(154)
	*O. fasciatus* (rock bream)	FasL	(155)

TRAIL (TNFSF10)	*Eptatretus burgeri* (inshore hagfish)	hgTRAIL	(156)
	*C. idella* (grass carp)	GC-TRAIL	(157)
	*D. rerio* (zebrafish)	Dr_TRAIL-like v1 Dr_TRAIL-like v2 Dr_TRAIL-like v3 Dr_TRAIL-like v4	(152)
	*O. mykiss* (rainbow trout)	Om_TRAIL-like	(41)
	*T. nigroviridis* (spotted green pufferfish)	Tn_TRAIL-like v1 Tn_TRAIL-like v2	(41)
	*Siniperca chuatsi* (mandarin fish)	SCTRAIL	(158)
	*T. rubripes* (Japanese pufferfish)	TRAIL-1 TRAIL-2 TRAIL-3	(133)

*The species and the name for each species gene are underlined text refers to cartilaginous fish species. References are also annotated*.

*N/A, non-available*.

Recombinant BAFF proteins have been generated in some of these fish species, such as, for example, the cartilaginous fish white-spotted catshark (129), and the teleost zebrafish (120), fugu (121), Japanese sea perch (122), yellow grouper (124), tongue sole (126), rock bream (128), or tilapia (127), to carry out functional studies. These studies have proven an increase on the number of leukocytes mediated by BAFF, although the authors did not clarify whether this was due to a promotion of cell survival or an increase on cell proliferation, and they did not demonstrate if the surviving/proliferating fish leukocytes were in fact B cells. In some of these studies, the authors tested the effect of recombinant BAFF on mouse splenic B cells co-stimulated with anti-IgM (120, 121, 124, 127, 129). In all these experiments, fish recombinant BAFF promoted the survival of mouse B cells, which indicates that the molecular mechanism underlying BAFF-mediated B cell survival is preserved throughout species, from cartilaginous fish to mammals. However, specific studies on the impact of BAFF on B cells from lower vertebrates are needed to reveal new aspects about the evolution of B cell homeostasis and activation. Recent studies from our group showed the role of BAFF on teleost B cells, using rainbow trout as a model (53, 159). In these studies, we determined that teleost BAFF recapitulated mammalian BAFF regulatory aspects on B cells. Teleost BAFF promoted the survival of B cells but did not induced significant proliferation, and also increased the levels of ASCs which consequently increased IgM secretion and upregulated the expression of surface MHC II, in a similar trend to that seen in mammals (132). The similarity between teleost and mammalian BAFF functions strengthens the hypothesis of BAFF as a key master regulator of B cell functionality throughout evolution. In mammals, BAFF is produced by macrophages, DCs, stimulated neutrophils, and at low levels by T cells, but is never produced by resting B cells (60, 160, 161). Strikingly, one of our findings was that not only myeloid cells produced BAFF but also that specific subsets of splenic and peritoneal B cells were able to produce BAFF in rainbow trout (53, 159). Interestingly, in mammals, B cells from B-cell chronic lymphocytic leukemia (162) or non-Hodgkin’s lymphoma (163), as well as B cells from patients with autoimmune disorders, namely, rheumatoid arthritis (164), systemic lupus erythematosus (165), and primary Sjogren’s syndrome (166) also express BAFF, which rescues them from apoptosis in an autocrine loop. Thus, our studies seemed to have revealed a primitive mechanism through which B cells would produce BAFF at the steady-state to regulate their homeostasis and function in teleost. It seems that this regulatory mechanism throughout evolution was later assumed by other immune myeloid cells, probably after the appearance of lymphoid follicles, although in mammals, it can reemerge in various B cell disorders such as autoimmune diseases (ADs) or B cell malignancies. In a parallel study, we were also able to report for the first time an upregulation of BAFF transcription in peritoneal IgM^+^ B cells from fish immunized intra-peritoneally (i.p.) with viral hemorrhagic septicemia virus (VHSV) (114). In addition, it has been very recently shown that *in vivo* transgenic overexpression of BAFF induced an increased production of IgD, IgM, and IgZ in zebrafish (167). This implies that BAFF is not only involved on the homeostasis of peripheral B cell compartments but also plays an important role on the activation of different B cell subsets present in teleost.

## A Proliferation-Inducing Ligand

Although APRIL is probably the most important B cell-regulating TNFSF together with BAFF, very little is known about fish APRIL. In fact, although BAFF homologs have been identified in many cartilaginous and bony fish (summarized in Table 3), only a few APRIL homologue sequences have been found in teleost, specifically in zebrafish, channel catfish, Atlantic salmon, rainbow trout (41), and grass carp (134) (Table 3). In addition, while BAFF is present in all vertebrates, APRIL is missing in cartilaginous fish, birds, and several bony fish (168). This has encouraged evolutionary immunologists to hypothesize that since BAFF and APRIL have structural and functional similarities and share receptors, the absence of APRIL on cartilaginous fish or the loss of APRIL in avian species could have been functionally compensated by BAFF. From the functional point of view, teleost APRIL has been shown to be mostly expressed in lymphoid tissues (spleen, head kidney) as well as in some mucosal tissues (skin, intestine) but at very low levels (41, 114, 134). After a bacterial or a viral challenge, the transcription of APRIL was quickly upregulated in immune tissues of grass carp (134), and recombinant APRIL promoted the survival of total splenocytes in zebrafish (169), indicating that this TNF ligand plays some role on the activation of the immune response. In addition, a study by our group showed that APRIL promoted the survival of peritoneal IgM^+^ B cells in rainbow trout (114). Since APRIL signaling through BCMA is key for the survival of PCs (70), it is tempting to hypothesize that those teleost species in which APRIL is found to have obtained a phenotypical advantage, the long-term survival of peripheral subsets of Ab-secreting PCs, that would have been positively selected.

## BAFF- and APRIL-Like Molecule (BALM)

Searching through the rainbow trout EST databases, Glenney and Wiens reported in 2007 the identification of an additional sequence with high similarity values with BAFF but containing a D–E loop characteristic of APRIL, which was subsequently designated BALM. This new TNFSF ligand was also found in fugu and three spined stickleback (41). This study also determined that BALM gene was mainly expressed in lymphoid tissues. In a more recent study, BALM orthologs have been found in cartilaginous fish (168) (Table 3), in addition to a BAFF-like sequence showing homology to BAFF, APRIL and BALM present in lampreys (168). In this study, the authors described that BALM is absent in all tetrapods, and there is a selective deletion of this gene in zebrafish. These data point to BALM as an ancestral BAFF-like ligand, which appeared early in evolution and was then lost when BAFF and APRIL acquired divergent functions. However, the presence of an ancient BAFF-like homolog in lampreys indicates that further research is needed to classify these closely related TNFSF members, and clarify their evolution and biological functions. From the functional point of view, it has been shown that teleost BALM can promote B cell survival and proliferation in rainbow trout kidney (170). Moreover, its transcription is upregulated on peritoneal lymphocytes after i.p. administration of VHSV (114), and it has also been demonstrated that there is a significant correlation between the expression of BALM and the progress of the proliferative kidney disease in rainbow trout (170). Although very little is yet known about this ligand, these data indicate that it plays a very important role in the activation of B cells on the responses against different types of pathogens in lower vertebrates.

## CD40L and OX40L

CD40L homologue sequences have been identified in several teleost species, such as rainbow trout, Atlantic salmon, zebrafish, fugu, and spotted green pufferfish (41) and more recently in one cartilaginous fish, the small-spotted catshark (131) (summarized in Table 3). In teleost, CD40L is mainly expressed in spleen, head kidney, and gills from resting animals (41, 171, 172). Furthermore, T cell mitogens such as PHA and ConA upregulated the transcription of CD40L on the spleen, head kidney, and gills of Atlantic salmon suggesting these may be special locations for T-B cell cooperation during TD immune responses (172). Interestingly, the production of CD40L by T cells was significantly increased in zebrafish immunized with TD Ags, and the upregulation of CD40L *in vivo* increased the levels of serum IgM on those animals (171). This last set of data by Yong-Feng Gong et al. elegantly demonstrated that the interaction between T and B cells through ligation of CD40 elicits TD immune responses in fish. Studies on the small-spotted catshark paralleled these results, showing that CD40L was mainly expressed in the spleen, as well as in the mucosal tissues, such as gills and gut (131). The transcription of CD40L was significantly upregulated after the addition of T cell polyclonal activators to cultures of PBL *in vitro*. Altogether, these data suggest that CD40L produced by T cells plays a very important role on TD immune responses. Since fish lack LNs and do not form lymphoid follicles, it would be of great interest to study how and where T–B cell interactions take place to better understand the evolutionary origins of TD immune responses.

To date, only one sequence with certain homology to OX40L has been reported in zebrafish (41), designated as Dr_TNF-New. This sequence was most similar to human OX40L and to *Xenopus* TNF-α. However, to the light of these results, it cannot be stated that true OX40L homologs exist in fish, and further studies are required its existence. In mammals, cross-linking of OX40L on B cells by OX40 expressed on activated CD4^+^ T cells promotes B cell proliferation (22). Since fish B cells seem to maintain many functions of innate B1 cells, and B–T cell interaction might be less abundant than that seen in mammals, it is tempting to hypothesize that the absence of OX40L in fish may be compensated by CD40L on the development of TD immune responses.

## TNF-α

TNF-α is probably the most studied TNFSF ligand, due to its potential roles in development, cell proliferation, apoptosis, inflammation, and immunity [reviewed in Ref. (173)]. Hirono et al. first described a fish homolog of TNF-α in the Japanese flounder (135). Since then, TNF-α homologs have been identified in a plethora of teleost species (summarized in Table 3), such as Japanese flounder (135), sea bream (139), channel catfish (140), turbot (143), sea bass (144), mandarin fish (145), striped trumpeter (148), tongue sole (150), Atlantic salmon (146), goldfish (147), bluefin tuna (149), common carp (141, 142), and rainbow trout (136–138).

Interestingly, several teleost species present multiple isoforms of TNF-α. Two copies were initially found in rainbow trout (137) and four within the common carp (141, 142). Given that both species are tetraploid, the presence of multiple TNF-α isoforms was not surprising. However, with the discovery of at least two TNF-α genes within non-tetraploid fish species, such as bluefin tuna (149), orange-spotted grouper (174), zebrafish, and medaka (151), it has become clear that teleost have two different groups of TNF-α genes. Furthermore, the analysis of the zebrafish and medaka genomes (151) showed that members from the two different groups of TNF-α genes were found on different chromosomes, with conserved genes around them, thus indicating that the presence of these two groups is a consequence of a duplication event that occurred within bony fish.

Although both groups of TNF-α molecules can be found in various fish genomes, the role played by them in the immune response remains to be determined. On those species containing a single copy, it has been shown that TNF-α is ubiquitously expressed in all tissues analyzed from unstimulated fish such as Japanese flounder, sea bream, or mandarin fish (135, 139, 145). Results regarding the activation of TNF-α expression after treatment with lipopolysaccharide (LPS) were consistent between species; as its expression on the head kidney was significantly upregulated after treatment with LPS both *in vitro* and *in vivo*. Although TNF-α expression was also upregulated in response to viral or bacterial infections, some differences between species have been observed. For example, in turbot, virus induced higher TNF-α expression than bacteria in kidney cells (although the response was shorter in time) (143), while a very recent study showed the opposite result in tongue sole kidney, spleen, and blood cells from virus or bacteria immunized virus (150). In any case, macrophage-driven inflammation was activated in both studies. This suggests that although TNF regulation seems to be very similar among fish species, the interaction between host and pathogen might shift the spatiotemporal expression of TNF-α, to adapt the response to each specific pathogen. A nice example of this can be seen in the three striped trumpeter, which in response to the infection with the ectoparasite *Chondracanthus goldsmidi* significantly upregulates the expression of TNF-α not only in the head kidney but also in the gills, the attachment site of the parasite (148), thus demonstrating the adaptation of the host response to different pathogens.

Regarding the species that present two copies of TNF-α, some differences were found for such isoforms on each species. Both TNF-α1 and TNF-α2 were constitutively expressed in a number of tissues of healthy Atlantic salmon (146). However, incubation of head kidney leukocytes with bacteria upregulated the expression of TNF-α2 but not TNF-α1, suggesting different activation pathways for each TNF molecule. In goldfish, TNF-α2 showed higher expression levels than TNF-α1, and TNF-α2 was also able to activate primary macrophages (147). Something similar was observed in bluefin tuna, where TNF-α2 mRNA was significantly higher than TNF-α1 in blood leukocytes (149). In addition, the expression of TNF-α2 was increased by stimulation with B cell, T cell, or macrophage activators, while the expression of TNF-α1 was not affected. These observations led the authors to propose that while TNF-α1 is a ubiquitous cytokine involved in cell survival and apoptosis, TNF-α2 is an inducible form which may be a key regulator of the innate immune response. In mammals, TNFR1 and TNFR2 exert different functions, being the former involved in cell death and the latter in the regulation of the immune response (15). Little is known about TNFR in fish, so it would be of great interest to study whether the different TNF-α isoforms found in fish signal through different receptors.

Of special interest is the case of the rainbow trout where an additional TNF-α isoform was found (138): TNF-α1 and TNF-α2 are expressed constitutively in some tissues, such as head kidney, and gills, and they induced a pro-inflammatory response on a rainbow trout macrophage cell line (137). By contrast, LPS induced a much faster, stronger, and longer upregulation of TNF-α2 expression when compared with TNF-α1, pointing to the fact that TNF-α1 could be involved in cell survival and apoptosis, while TNF-α2 is an inducible form involved in the activation of the immune response. Moreover, the third form (TNF-α3) identified, showed low identity with the two forms previously described (138). Its basal expression was the lowest among the three molecules, but it was the most responsive of them against different stimuli, showing a strong upregulation of its expression at very early time points after stimulation of primary leukocytes with LPS or T cell mitogens. In addition, TNF-α3 induced the expression of many pro-inflammatory mediators and immune regulators (138). This suggests that TNF-α3 might be in fact a strong amplifier of the early inflammatory and immune responses.

In mammals, TNF-α has been proven as an important co-stimulator of B cells for their polyclonal expansion on primary immune responses (15). Fish B cells retain many innate immune features, such as phagocytic capacities (115) and expression of multiple TLRs (119), and their responses are likely to be TI since no GCs are observed. Thus, fish B cells responses seem to be based in polyclonal activation of Ag-reactive pools of cells (175). Since the direct effect of recombinant TNF-α has not been tested on fish B cells, we think it is paramount to study if this TNFSF ligand can improve B cell-mediated Ab responses.

## LTβ

Initially, homologous sequences of mammalian LTβ were found in zebrafish and fugu (142), designated as TNF-new (TNF-N). Posterior investigations identified homologs of this gene in medaka and zebrafish (151). Phylogenetic analysis of these sequences with known vertebrate sequences showed a closer relationship of these sequences to TNFSF3 (LTβ) than to TNFSF1 and TNFSF2. This was further supported with the inclusion of *Xenopus* sequences, which are available for all TNFSF1, 2, and 3 (50). Interestingly, in rainbow trout, two different isoforms were isolated, and named LT-β1 and LT-β2 (50) (Table 3). The authors hypothesized that the presence of two isoforms was a result of the additional genome duplication event that took place in salmonids around 400 million years ago (176, 177). LT-β1 was expressed in the spleen, head kidney, intestine, and gills, while LT-β2 was expressed only in the gills of rainbow trout (50). This might indicate a different role for each LTβ in the homeostasis of B and T cells. In mammals, LTβ has been involved in the formation of LN, organization of lymphoid structures, and the formation of GCs (81). The absence of LN or GC reactions in fish may explain why LTβ has not yet been identified in other cartilaginous or bony fish species. Nevertheless, immunological assays are required to clarify whether this cytokine is needed in fish, as well as its potential role in central and peripheral immune tissues in rainbow trout, the only fish in which the presence of LTβ has been shown to date (50).

## Fas Ligand

Fas ligand is a well-known TNFSF ligand that controls the extrinsic apoptosis pathway (178). In fish, homologue sequences of FasL have been reported in some teleost, namely, zebrafish (152), rainbow trout (41), Japanese flounder (153), Nile tilapia (154), rock bream (155), and Japanese pufferfish (*Takifugu rubripes*) (133) (Table 3). Homologue sequences have also been identified in cartilaginous fish (52). In all the species studied, FasL was highly expressed on spleen and head kidney, which are secondary lymphoid tissues in fish. In some species, such as rainbow trout and tilapia, it was also expressed on the gills and gut mucosal tissues (41, 154). Functional studies have determined that the T cell mitogen PHA was able to upregulate the expression of FasL in PHA-bound lymphocytes from Japanese flounder (153) while LPS was not able to do so, thus showing that activated T cells are responsible for production of FasL. In line with this, mammalian FasL has been widely reported to be expressed in Ag-activated CD8^+^ and CD4^+^ T cells and natural killer cells (80). A similar upregulation of FasL has been observed in rock bream after a viral challenge, suggesting that FasL may also play a role on the regulation of antiviral immune responses (155). On the other hand, Fas is expressed at low levels on resting B cells but is upregulated after Ag activation (79), making these cells more susceptible to FasL-mediated cell death. Thus, it has been proposed that FasL–Fas interaction controls the size of the B cell compartment in homeostasis, while during the immune response it seems to be responsible for the elimination of activated B cells after the generation of ASCs and memory B cells, and the subsequent clearance of the pathogen (179). Recombinant tilapia FasL has been shown to induce apoptosis on Fas-expressing HeLa cell lines (154), demonstrating its functionality. Unfortunately, the effect of FasL on fish B cells has not yet been addressed, so further investigations are needed to understand the impact of FasL on the B cell compartment in physiological or pathological conditions.

## TNF-Related Apoptosis-Inducing Ligand

TNF-related apoptosis-inducing ligand sequences have been characterized in several teleost fish, such as grass carp (157), zebrafish (152), rainbow trout (41), pufferfish (41), and mandarin fish (158) (summarized in Table 3). In humans, there is only a single TRAIL gene while two genes have been reported in avian species (41). Eimon et al. identified four TRAIL genes in zebrafish (41, 152). Hence, the authors proposed that the ancestral gene was duplicated before the divergence of ray-finned and lobe-finned fishes (around 400 million years ago) (152). In line with this, three genes with homology to TRAIL have been identified in fugu (133). This study also showed that each of these homologs presented a very different gene organization forming distinct groups (133). In a thorough study based on the search of immune genes in leukocytes from jawless fish, a sequence showing homology to TRAIL was identified in the inshore hagfish (*Eptatretus burgeri*) (156), pointing to a conserved role of this cytokine throughout evolution. In all the teleost fish analyzed, TRAIL was expressed mainly in spleen and kidney but also in mucosal tissues, such as gills, skin, and/or intestine, depending on the species. In zebrafish, overexpression of TRAIL has been reported to induce cell death by activation of the extrinsic apoptosis pathway machinery (152). In parallel, mandarin fish recombinant TRAIL-induced apoptosis of HeLa cells (158) although it was previously reported that the extracellular domain of the death receptors (DR) for TRAIL in zebrafish differs from those in human DR4 and DR5 (152), suggesting that cross-reactivity with mammalian DRs could only be achieved with certain fish TRAIL proteins. In mammals, TRAIL is involved in B cell differentiation at the GC reaction, and although fish lack secondary lymphoid structures and do not form GCs, it would be of great interest to investigate what is the role of TRAIL on B cells from lower vertebrates.

## Concluding Remarks

A plethora of studies in mammals have revealed that TNF ligand–receptor interactions elicit complex and divergent functions during the immune response. TNFSF ligands have the ability to induce both cell death or cell activation/co-stimulation and although some of these molecular mechanisms have been elucidated, the origin of these complex interactions and their multiple functionalities are not yet fully understood. TNFSF ligands play a key role in the immune response, and many researchers have taken advantage of this to develop new therapeutic strategies based on the modulation of TNF ligands. In fact, several anti-TNF neutralizing Abs are already available or under clinical trials for the treatment of inflammatory and ADs. Moreover, many immunologists are considering the option of using engineered TNFSF ligands as vaccine adjuvants. For instance, DNA vectors encoding for CD40L multi-trimers are being tested as adjuvants for a HIV vaccine, based on their positive effects on B cell co-stimulation during the vaccination process, thus generating high-affinity Abs against the virus. Hence, this is a fascinating research topic that is becoming of great importance within the field of immunotherapy. Most of the questions that have not been answered yet could be explained through the analysis of the evolution of the TNF superfamily of ligands and receptors. From an evolutionary point of view, it is widely accepted that acquired immunity first appeared in fish. This animal taxon possesses unique immune features, and recent evidences suggest that we can learn much about the evolution and functionality of TNF ligands in fish. For instance, they retain a unique TNF cytokine, BALM, which has become extinct in tetrapods, and although its function is still unknown, many evidences point to the fact that this molecule could be key to understand the molecular and functional divergence of BAFF and APRIL. On the other hand, fish express several isotypes of some TNF ligands (i.e., TNF-α) while mammals only express one. In fish, these ligand isotypes show different expression patterns, activation profiles, and immune functions. Thus, analyzing these isotypes could provide us with vital information about their original function, or about how and why some members of the TNF family were lost or acquired throughout evolution. Moreover, this analysis could also help to elucidate the convergence or divergence of some immune functions played by TNF ligands.

## Author Contributions

CT and AG reviewed the bibliography and wrote the manuscript.

## Conflict of Interest Statement

The authors declare the absence of any commercial or financial relationships that could be construed as a potential conflict of interest.

## Abbreviations

Ab, antibody; AD, autoimmune disease; Ag, antigen; APRIL, a proliferation-inducing ligand; BAFF, B cell-activating factor of the TNF family; BAFFR, BAFF receptor; BCMA, B cell maturation antigen; BCR, B cell receptor; Blimp-1, B lymphocyte-induced maturation protein-1; BM, bone marrow; CRD, cysteine-rich domain; CSR, class-switch recombination; DcR3, decoy receptor 3; DR3, death receptor 3; DC, dendritic cell; DD, death domain; EDA, ectodysplasin; EDAR, EDA receptor; FasL, Fas ligand; GC, germinal center; GITR, glucocorticoid-induced TNFR; GITRL, GITR ligand; HVEM, herpesvirus entry mediator; LPS, lipopolysaccharide; LIGHT, homology with lymphotoxin, inducible expression, competing for GpD of herpes virus, that binds to the HVEM, and is expressed on activated T lymphocytes; LN, lymph node; LT, lymphotoxin; MHC, major histocompatibility complex; MZ, marginal zone; NGFR, nerve growth factor receptor; NHL, non-Hodgkin’s lymphoma; NK, natural killer; PC, plasma cell; RA, rheumatoid arthritis; RANK, receptor activator of NF-κB; RANKL, RANK ligand; RELT, receptor expressed in lymphoid tissues; SHM, somatic hypermutation; SLE, systemic lupus erythematosus; SS, Sjögren’s syndrome; TACI, transmembrane activator and calcium-modulating cyclophilin ligand interactor; TCR, T cell receptor; TD, thymus dependent; THD, TNF homology domain; TI, thymus independent; TLR, toll-like receptor; TNF receptor superfamily, tumour necrosis factor receptor superfamily; TNF ligand superfamily, tumour necrosis factor (ligand) superfamily; TRAIL, TNF-related apoptosis-inducing ligand; TWEAK, TNF-like weak inducer of apoptosis and proliferation.
